# SOCS Proteins as Regulators of Inflammatory Responses Induced by Bacterial Infections: A Review

**DOI:** 10.3389/fmicb.2017.02431

**Published:** 2017-12-12

**Authors:** Skyla A. Duncan, Dieudonné R. Baganizi, Rajnish Sahu, Shree R. Singh, Vida A. Dennis

**Affiliations:** Center for NanoBiotechnology Research, Alabama State University, Montgomery, AL, United States

**Keywords:** SOCS, immune response, inflammation, bacteria, cytokines, JAK/STAT, therapy, signaling

## Abstract

Severe bacterial infections can lead to both acute and chronic inflammatory conditions. Innate immunity is the first defense mechanism employed against invading bacterial pathogens through the recognition of conserved molecular patterns on bacteria by pattern recognition receptors (PRRs), especially the toll-like receptors (TLRs). TLRs recognize distinct pathogen-associated molecular patterns (PAMPs) that play a critical role in innate immune responses by inducing the expression of several inflammatory genes. Thus, activation of immune cells is regulated by cytokines that use the Janus kinase/signal transducers and activators of transcription (JAK/STAT) signaling pathway and microbial recognition by TLRs. This system is tightly controlled by various endogenous molecules to allow for an appropriately regulated and safe host immune response to infections. Suppressor of cytokine signaling (SOCS) family of proteins is one of the central regulators of microbial pathogen-induced signaling of cytokines, principally through the inhibition of the activation of JAK/STAT signaling cascades. This review provides recent knowledge regarding the role of SOCS proteins during bacterial infections, with an emphasis on the mechanisms involved in their induction and regulation of antibacterial immune responses. Furthermore, the implication of SOCS proteins in diverse processes of bacteria to escape host defenses and in the outcome of bacterial infections are discussed, as well as the possibilities offered by these proteins for future targeted antimicrobial therapies.

## Introduction

Cytokines are signaling molecules secreted by cells to elicit a particular effect on the behavior and communication of surrounding cells (Dinarello, 2000, 2007; Zhang and An, 2007). They are known protagonists in the development and pathology of a variety of diseases, including but not limited to, autoimmune (He et al., 2016), rheumatoid arthritis (Khondker and Khan, 2014), celiac (Girard-Madoux et al., 2016), bacterial (Yilma et al., 2013), Crohn's (Smith et al., 2009), and cystic fibrosis (Dosunmu et al., 2016). Cytokines are either pro-inflammatory (e.g., IL-6, IFN-γ, TNF-α, IL-1β), anti-inflammatory (e.g., IL-10, IL-1RA, IL-4, IL-13) or chemokines (e.g., IL-8, CCL2, CCL5, CXCL1, CXCL10). While pro-inflammatory cytokines help to exacerbate disease and are algesic (Uceyler et al., 2009), anti-inflammatory cytokines are analgesic (Uceyler et al., 2009) and promote healing, while reducing inflammation. Chemokines are immune migration factors that stimulate the recruitment of leukocytes to the sites of infection. Research evidence has shown that some cytokines participate in both the initiation and persistence of pathologic pain by directly activating nociceptive sensory neurons, which respond to potentially harmful stimuli such as sprains, bruises, burns, and inflammation (Uceyler et al., 2009). Furthermore, pro-inflammatory cytokines (e.g., IL-1β, TNF-α) (Copray et al., 2001; Ozaktay et al., 2006) and chemokines (e.g., CCL2) (Oh et al., 2001; White et al., 2005) may directly modulate neuronal activity in the peripheral and central nervous systems (Zhang and An, 2007).

The breadth, persistence and robust nature of immune responses are dictated by the integration of complex immune signaling cascades mediated by TLRs along with B-cells, T-cells and cytokine receptors (Elliott and Johnston, 2004; Dinarello, 2007). During an immune response, positive signals sent to immune cells via signaling pathways get activated by effector and regulatory T-cells using their negative feedback mechanisms (Dinarello, 2007). This ability of cytokines to have both positive and adverse effects on the immune system highlights the complexity in solidifying the exact role of cytokine biology to structure and function ratio. Innate immune responses although necessary for host survival also may be associated with adverse disease pathology. For example, IFN-γ is essential for defense against several intracellular bacteria such as *Listeria monocytogenes, Francisella tularensis, Mycobacteria tuberculosis*, and *Chlamydia trachomatis* but yet bolsters the pathogenesis of several autoimmune diseases (Huang et al., 1993; Harty and Bevan, 1995; Dinarello, 2007). Also, despite the fact that IL-2 is crucial for the generation of cytotoxic T-cells (CTLs) and forms the basis for several vaccines, it drives graft vs. host disease and limits the success of bone marrow transplantation (Dinarello, 2007). Understanding when and how cytokines illicit their pleiotropic and redundant effects on immune responses are essential for designing effective drug therapies.

Suppressor of cytokine signaling (SOCS) family of proteins apparently are modulators of a variety of diseases including those with autoimmune etiologies, inflammation, allergies, bacteria, and cancer. SOCS regulate signaling pathways on an intracellular level to potently and specifically inhibit cytokine and growth factor signaling (Yoshimura et al., 2005; Linossi et al., 2013; Ushiki et al., 2016). There are eight related SOCS family of proteins [SOCS 1-7 and CIS (cytokine-inducible SH2-containing protein)] (Masuhara et al., 1997; Trengove and Ward, 2013; Hao and Sun, 2016) that regulate cytokine signaling by inhibiting JAK activity or targeting signaling components for ubiquitination. Studies have revealed that SOCS protein expression induced by cytokine stimulation can negatively impede cytokine signaling by blocking the JAK/STAT pathway (Cooney, 2002; Elliott and Johnston, 2004; Croker et al., 2008; Tamiya et al., 2011). Other stimuli, including lipopolysaccharide (LPS), bacterial products, and chemokines can also induce SOCS expression (Rakesh and Agrawal, 2005). Since cytokines primarily regulate host immune responses to infection, the tight modulation of cytokines release may hinder disease progression. This review will delve into the regulation of several key cytokines or cytokine cascades by the central action of the intracellular SOCS proteins during a bacterial-induced inflammatory response. Emphasis will be placed on the mechanisms involved in SOCS proteins induction and regulation of antibacterial immune responses. Furthermore, the implication of SOCS proteins in diverse processes of bacteria to escape host defenses and in the outcome of bacterial infections are discussed, as well as the possibilities offered by these proteins for future targeted antimicrobial therapies.

## SOCS family of proteins and regulation of immune response

### Structure of the SOCS box as related to function

The SOCS protein structure consists of an N-terminal domain, a central SH2 domain and a C-terminal SOCS box (Bullock et al., 2007; Hao and Sun, 2016). They all share sequence homology, but especially these pairs, CIS/SOCS1/SOCS2, SOCS3/SOCS4/SOCS5, and SOCS6/SOCS7 have unquestionable marked pair-wise homology. Specifically, the SOCS box is a small, 40- to 60-amino acid (aa) residue domain structurally similar to the domain of the von Hippel–Lindau protein and lesser to the F-box from Skp2 (Kile et al., 2002). The SOCS box interacts with Elongins (B and C) to recruit E2 ubiquitin–transferase, necessary for negative regulation of cytokine signaling (Kamizono et al., 2001). The interaction between SOCS and Elongin BC complex and Cullin 2, facilitates the ubiquitination of JAKs and their cytokine receptors, which targets them for proteasomal degradation (Rawlings et al., 2004; Kershaw et al., 2013).

Structurally, SOCS family of proteins can be subdivided based on aa residues, with the shortest N-terminal region being CIS, SOCS1-3, or longest being SOCS4-7. CIS and SOCS1-3 act in a negative feedback loop through the JAK/STAT pathway in response to cytokine signaling; whereas, SOCS4-7 mainly regulate growth factor receptor signaling (Krebs et al., 2002; Kario et al., 2005; Trengove and Ward, 2013) (Table 1). Notably, SOCS1 and SOCS3 share a similar kinase inhibitory region (KIR) at the N-terminus that is essential for JAK inhibition (Sasaki et al., 1999; Yasukawa et al., 1999; Alexander, 2002; Ushiki et al., 2016). The SH2 domain/KIR ability to inhibit the signaling cascades independently by either blocking STAT docking or directly inhibiting JAK kinase activity confers substrate specificity. Depending on the size and structure of the SOCS protein, each domain interacts directly or indirectly with JAKs or their specific cytokine receptors to inhibit signaling proteins (Hilton, 1999; Nicholson et al., 1999; Sasaki et al., 1999, 2000; Yasukawa et al., 1999; Lehmann et al., 2003). Supposedly, the SOCS box mediates signaling suppression differently by promoting the degradation of bound signaling intermediates via an interaction with the cellular ubiquitination machinery (Zhang et al., 1999, 2001; Kamizono et al., 2001; Kile et al., 2002; Rui et al., 2002; van de Geijn et al., 2004). Revealing how SOCS proteins associate and interact with other proteins or external factors may offer much-needed premise in biomedical therapy approaches.

**Table 1:** The functions of SOCS 1-7 and CIS proteins.

**SOCS Proteins**	**Functions**
SOCS 1	•Regulates M1-macrophage activation by inhibiting the interferon gamma-induced JAK2/STAT1 pathway and TLR/NF-κB signaling (Frobose et al., 2006; Zhou et al., 2010). •Regulates M2 macrophage polarization (Frobose et al., 2006). •Tumor suppressor (Met receptor inhibition and enhancement of p53 tumor suppressor activity) (Gingras et al., 2004).
SOCS 2	•M2 polarization and limits M1 polarization (Frobose et al., 2006). •Feedback inhibitor of TLR-induced activation in dendritic cells (Frobose et al., 2006).
SOCS 3	•Negative regulation of cytokines that signal through the JAK/STAT pathway (Lehmann et al., 2003; Carow et al., 2013). •Inhibits cytokine signal transduction by binding to tyrosine kinase receptors including gp130, LIF, erythropoietin, insulin, IL12, GCSF and leptin receptors. •Binding to JAK2 inhibits its kinase activity. •Suppresses fetal liver erythropoiesis. •Regulates onset and maintenance of allergic responses mediated by T-helper type 2 cells. •Regulates IL-6 signaling *in vivo* (By similarity). Probable substrate recognition component of a SCF-like ECS (Elongin BC-CUL2/5-SOCS-box protein) E3 ubiquitin-protein ligase complex which mediates the ubiquitination and subsequent proteasomal degradation of target proteins.
SOCS 4-6	•Regulate epidermal growth factor (EGF) signaling.
SOCS 7	•Regulates signaling cascades probably through protein ubiquitination and/or sequestration. •Functions in insulin signaling and glucose homeostasis through IRS1 ubiquitination and subsequent proteasomal degradation. •Inhibits prolactin, growth hormone and leptin signaling by preventing STAT3 and STAT5 activation, sequestering them in the cytoplasm and reducing their binding to DNA. •Mediates the interaction with the Elongin BC complex, an adapter module in different E3 ubiquitin ligase complexes (By similarity).
CIS	•Negative regulation of cytokines that signal through the JAK/STAT5 pathway such as erythropoietin, prolactin and interleukin 3 (IL3) receptor (Mui et al., 1996; Sasi et al., 2014; Tobelaim et al., 2015). •Inhibits STAT5 trans-activation by suppressing its tyrosine phosphorylation (Chretien et al., 1996; Matsumoto et al., 1997). •May be a substrate-recognition component of a SCF-like ECS (Elongin BC-CUL2/5-SOCS-box protein) E3 ubiquitin-protein ligase complex which mediates the ubiquitination and subsequent proteasomal degradation of target proteins (Yoshimura, 1998).

### SOCS signaling pathway

The SOCS proteins were first identified based on their ability to suppress cytokine signaling through the JAK/STAT pathway (Dalpke et al., 2003, 2008). The mechanism of cytokines binding to their putative cell surface receptors induces receptor dimerization, which allows trans-phosphorylation of JAKs (Dalpke et al., 2008) and tyrosine phosphorylation of the intracellular receptor subunits, to be bound by STATs. Following STAT phosphorylation, there is dimerization and then translocation into the nucleus (Dalpke et al., 2001). All SOCS proteins inhibit the JAK/STAT pathway similarly, (Dalpke et al., 2001; Caballero et al., 2016) upon cytokine stimulation, which blocks further signaling in a classic feedback loop by targeting signaling intermediates for degradation (Elliott and Johnston, 2004). Moreover, SOCS proteins have been implicated in regulating inflammation and determining cell fate because their obstruction or imbalance causes a broad range of diseases (Elliott and Johnston, 2004).

Upon receiving a signal, a receptor protein changes conformation simultaneously, creating a series of biochemical reactions within the cell that are amplified by intracellular signaling pathways. The JAK/STAT pathway, which coincidentally is involved in SOCS induction, serves as the primary signaling mechanism for most cytokines in mammals (Rawlings et al., 2004). Moreover, the JAK/STAT circuitry includes a negative feedback loop that activates STATs to stimulate the transcription of SOCS genes (Alexander, 2002; Rawlings et al., 2004). JAK is first activated when various ligands, usually cytokines and growth factors bind to cell surface receptors to form a dimer that can phosphorylate each other. This phosphorylation further activates JAK, allowing it to phosphorylate the receptor. When STAT binds to the receptor, it then becomes phosphorylated by JAK. Once phosphorylated, STAT dimerization occurs followed by translocation to the nucleus, where it binds to specific sequences in the DNA. Inactivation of STATs occurs via dephosphorylating proteins along the signaling pathway. Alterations or mutations that perturb the JAK/STAT pathway will affect homeostasis, growth regulation, survival and cell migration; which are all critical functions of this pathway (Rawlings et al., 2004). Furthermore, mutations that activate or fail to regulate JAK signaling properly, cause inflammatory diseases and other etiologies (Rawlings et al., 2004). Because understanding the mechanism of signaling during SOCS-induced responses to bacteria can assist in halting or altering disease pathogenesis, this pathway is of great scientific interest for targeted therapeutics.

## Bacterial pathogenesis and immune response

### Bacterial pathogenesis

Despite this new era of biomedical development, the leading cause of mortality is still significantly influenced by new and pre-existing infectious diseases (O'Connor et al., 2006). Added to this for further exacerbation is the increasing incidence of antimicrobial resistant strains, the emergence of new diseases, and the re-surging of older deadly infectious diseases causing a direct negative impact on the economy and welfare in endemic areas (Peterson, 1996; Morens et al., 2004). It is well-documented that microbial pathogens use common strategies to cause infection and disease. These include adherence, invasion, and enhanced pathogenicity, while also evading host defenses (Peterson, 1996; Wilson et al., 2002; Morens et al., 2004). A common strategy employed by bacterial pathogens is the Type III secretion system (T3SS), which in some cases can be used to invade host cells and/or evade immune detection by injecting bacterial signaling proteins to manipulate host immune response for their intracellular survival. Some bacteria that employ the T3SS machinery, as well as the secreted effector proteins for their virulent functions, are *C. trachomatis* (Betts-Hampikian and Fields, 2010), *Yersinia pestis* (Nair et al., 2015)*, Salmonella serovar* Typhi (Johnson et al., 2017)*, Shigella* (Hu et al., 2017)*, Escherichia coli* (Hu et al., 2017; Shaulov et al., 2017), and *Pseudomonas aeruginosa* (Brannon et al., 2009). Pathogens may also reside within a phagolysosome, a phagosome or within the host cell cytosol to evade host immune responses (Wilson et al., 2002). The production of virulent microbial toxins also plays a vital role in the pathogenesis of some diseases (de Sousa, 2003; Ramachandran, 2014). Highly infectious microbes such as *Clostridium tetani* (tetanus toxin) (Caballero et al., 2016), *Corynebacterium diphtheria (*diphtheria toxin) (Bermejo-Martin et al., 2016), *Shigella dysenteriae* (Shiga toxin) (Zadravec et al., 2016), and *Clostridium botulinum* (botulinum toxin) (Ozcan and Ismi, 2016) produce some of the most potent and lethal toxins.

### Immune responses to bacterial infections

The manifestation and severity of a disease are under the influence of the host immune response induced by a bacterial pathogen. Mediation of the host defense mechanisms occurs by its primary and secondary defense responses, respectively innate and adaptive immune responses (Chaplin, 2010). Consequently, the host inflammatory response may be the most important for dealing with microbial infections because it purposely diverts antimicrobial factors such as phagocytes and lymphocytes directly to the infection site. Mediation of inflammation occurs via central effector cells such as mast cells or blood basophils that give rise to localized or systemic responses, respectively (Chaplin, 2010; Ren and Dubner, 2010). Other effectors include phagocytes that engulf microbes, neutralization of microbial pathogens by antibodies or toxins that possess potent antimicrobial properties as well as by lymphocytes and macrophages that initiate immune responses against the pathogen (Tosi, 2005).

When bacteria, such as *Neisseria meningitidis*, and *Salmonella* spp. invade their respective hosts; complement proteins are up-regulated and assist in bacteria-killing via complement-mediated lysis (Finlay and McFadden, 2006; Lewis and Ram, 2014). Gram-positive bacteria such as *Staphylococcus* spp. that are resistant to this type of bacteria-killing mechanism eventually will become opsonized by acute phase proteins and destroyed by phagocytes. However, other pathogens can avoid these above-described killing mechanisms. In these cases, the host relies on cell-mediated immune responses to identify and eliminate such organisms. Macrophages are targets for intracellular bacteria (e.g., *Salmonella* spp.) that have evaded detection by complement or antibody (do Vale et al., 2016). When infected, these macrophages use MHC class II molecules to present bacterial peptides on their cell surface for recognition by T-helper cells (Goldman and Prabhakar, 1996). T-helper cells recognize the microbial peptides and release IFN-γ that initiates killing mechanisms for clearance of the invading intracellular bacterium (Goldman and Prabhakar, 1996). Notably, many bacteria can benefit from the stimulation of inflammatory reactions as their induced responses usually cause considerable tissue damage to the host making the host more susceptible to an infection (Mogensen, 2009). Moreover, the same cytokines and chemokines present at the inflammatory site are also very critical in regulating the immune system and inflammation (Cekici et al., 2000). Thus, dysregulation or an improper balance of cytokine signaling can cause a variety of diseases not only limited to bacterial but also including allergy, intensified inflammation, and some forms of cancer (26). It is therefore urgent that additional studies be performed with SOCS proteins as inflammatory regulators to encourage novel therapeutic approaches to eradicate bacterial diseases.

### Broad activity of SOCS proteins in bacterial responses

Robust innate and adaptive immune responses against microbial pathogens are determined by the detection of the diverse repertoire of their specific PAMPs, by PRRs of the host innate immune cells such as TLRs, and nucleotide oligomerization domain proteins (NOD) (Janeway and Medzhitov, 2002; O'Riordan et al., 2002; Takeuchi and Akira, 2010). Upon bacterial infection and PAMPs recognition, the PRRs initiate highly complex intracellular signaling pathways, which trigger pro-inflammatory and antimicrobial responses allowing the host to respond promptly to the infection (Athman and Philpott, 2004; Philpott and Girardin, 2004; Kumar and Yu, 2006; Gerold et al., 2007; Mogensen, 2009; Takeuchi and Akira, 2010; Stokes et al., 2015). TLRs play a central role in recognition of PAMPs and in driving host inflammatory responses. They activate the cells of innate immunity and promote pathogen-specific adaptive immunity through their action on antigen-presenting cells (Dalpke et al., 2001; Athman and Philpott, 2004; Kumar and Yu, 2006; Tapping, 2009). Triggering of PRRs and cytokine signaling in immune effector cells induces the expression of inflammatory and antimicrobial mediators as well as regulatory factors, which coordinate the elimination of the pathogen and infected cells (Mogensen, 2009; Takeuchi and Akira, 2010; Stokes et al., 2015). This process mainly occurs via the activation of JAK/STAT signaling pathways and results in gene expression and production of a variety of molecules, including an array of cytokines, chemokines, growth factors and immune-receptors (Rawlings et al., 2004; Mogensen, 2009). These proteins, especially cytokines play essential roles as mediators of immune responses and therefore have to be tightly regulated to induce appropriate and safe antimicrobial responses (Baetz et al., 2007; Dalpke et al., 2008).

SOCS proteins, protein inhibitors of activated stats (PIAS) and protein tyrosine phosphatases (PTPs) are negative regulators that activate the JAK/STAT pathway effectors of PRRs (Rawlings et al., 2004; Abbas et al., 2012). SOCS proteins represent one of the fundamental molecular mechanisms, which regulate the level of microbial pathogen-induced signaling of cytokines employing JAK/STAT signaling cascades (Yoshimura et al., 2012; Trengove and Ward, 2013; Kyoko Inagaki-Ohara, 2014), and also they interfere with cell signaling by mediating the degradation of signaling proteins (Grutkoski et al., 2003). These proteins regulate a broad range of pro- and anti-inflammatory cytokines in immune cells and therefore determine the sensitivity of the host to bacterial infections and the outcome of various bacterial infections (Dalpke et al., 2003; Baetz et al., 2004; Takagi et al., 2004; Yoshimura et al., 2004, 2007; Chaves de Souza et al., 2013).

### Gram-negative bacteria and SOCS proteins

**Figure 1:**
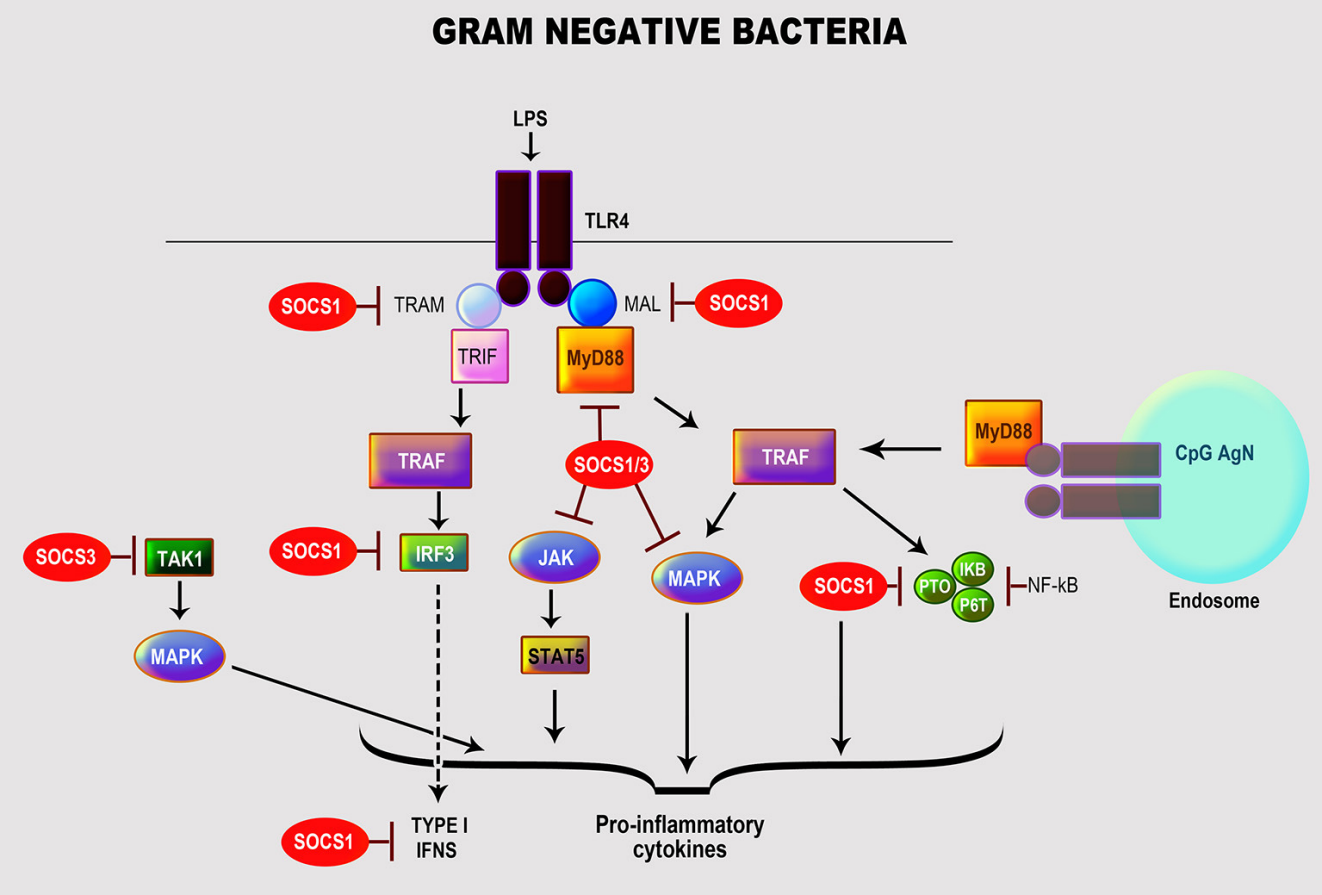
Role of SOCS proteins in the regulation of the signaling pathways induced by recognition of gram-negative bacteria. Recognition of gram-negative bacteria through LPS by TLR4. Activation by LPS of TLR4 leads to the activation of transcription factor NF-κB and MAP kinases (JNK, p38, ERK) by myeloid differentiation factor 88 (MyD88)-dependent pathway, serine/threonine kinase IL-1R-associated kinase 4 (IRAK-4), and TNFR-associated factor 6 (TRAF-6) resulting in the induction of essential cytokines and chemokines (Nakagawa et al., 2002; Qin et al., 2007; Freudenberg et al., 2008; Hu et al., 2009). The regulation of TLR signaling by the specific SOCS protein involved is highlighted in red.

Many gram-negative bacteria of the genera *Escherichia, Pseudomonas, Chlamydia, Klebsiella, Neisseria*, and *Salmonella* can cause spectra of manifestations in humans (Kang et al., 2005; Mogensen, 2009). Their cell wall is composed of peptidoglycan surrounded by LPS, phospholipids, and proteins (Mogensen, 2009); LPS is their main immune-stimulatory component and primary PAMP (Freudenberg et al., 2008). LPS interacts with host immune cells via TLR4 in association with several co-receptors: myeloid differentiation protein-2 (MD2), CD14 and LPS-binding protein (LBP) (Dumitru et al., 2000; Kumar and Yu, 2006; Strengell et al., 2006; Freudenberg et al., 2008). Moreover, these bacteria can simultaneously activate other TLRs via alternative PAMPs, including TLR2 (peptidoglycan and bacterial membrane proteins), TLR9 (non-methylated CpG (cytosine-guanosine)-DNA), and TLR5 (flagellin) (Mogensen, 2009). The interaction of LPS with TLR4 leads to activation of NF-κB and MAPK (JNK, p38, ERK) via myeloid differentiation factor 88 (MyD88)-dependent pathway, serine/threonine kinase IL-1R-associated kinase 4 (IRAK-4), and TNFR-associated factor 6 (TRAF-6). Besides, there are MyD88-independent pathways that activate interferon regulatory factor-3 (IRF-3) and IRF-7 resulting in the induction of IFN-dependent genes to activate the JAK/STAT pathway (Qin et al., 2007; Freudenberg et al., 2008; Hu et al., 2009). These various activation machineries culminate in triggering multiple immune response genes, especially pro-inflammatory cytokines and chemokines (Nakagawa et al., 2002; Qin et al., 2007; Freudenberg et al., 2008; Hu et al., 2009) (Figure 1).

Host immune cells have developed negative regulatory mechanisms, such as SOCS proteins, to control the exacerbated inflammatory reactions caused by prolonged exposure to LPS. Studies have shown that SOCS1 protects a host from fatal LPS responses (Kinjyo et al., 2002; Nakagawa et al., 2002; Hu et al., 2012), as underscored in SOCS1-deficient mice that exhibit a high sensitivity to LPS mediated thru MyD88-dependent and—independent pathways in association with IRAK1 (Kinjyo et al., 2002; Baetz et al., 2004; Croker et al., 2008; Manicassamy and Pulendran, 2009; Fujimoto and Naka, 2010). SOCS1 also facilitates blocking the uptake of LPS in mouse hepatocytes potentially to control sepsis (Scott et al., 2009). Experiments using SOCS1- and IFN-γ-deficient mice showed that IFN-signaling was modulated via JNK, p38, and NF-κB activations (Kinjyo et al., 2002; Croker et al., 2008) through direct interactions with NF-κB p65 and TLR/MAL (MyD88-adaptor-like protein) leading to their suppression and degradation (Nakagawa et al., 2002; Abbas et al., 2012). Others have reported that SOCS1 regulates the IFN-β-induced JAK/STAT pathway by directly inhibiting STAT1 phosphorylation and indirectly TLR4 signaling via IRF-3 (Wilson, 2014).

SOCS3 plays a vital role in regulating LPS inflammation by targeting multiple cytokine signaling cascades. Results from Qin et al. (2007) confirm that the transcriptional expression of SOCS3 by LPS in macrophages and microglia was mediated by activation of MAPK (ERK1/2, JNK, p38), STAT3 and endogenously produced IL-10. Macrophages deficient in SOCS3 expressed heightened LPS-induced STAT1, STAT3, and IL-6, but with no ensuing effect on NF-κB and ERK1/2 activation (Qin et al., 2012; Wilson, 2014). Moreover, it appears that depletion of SOCS3 in macrophages results in positively regulating TLR4 responses by, respectively suppressing STAT3- and SMAD3-mediated IL-6R and TGF-β activations, which are both necessary for negatively regulating LPS-induced IL-6 and TNF-α (Frobose et al., 2006). Also, SOCS3 has been implicated in controlling bone-associated inflammation as it inhibited LPS-induced IL-6 in osteoblasts by blocking the transcription factor, CAAT/enhancer-binding protein (C/EBPβ) (Yan et al., 2010). Paradoxically, SOCS3 positively regulated LPS/TLR4 responses by a feedback inhibition of endogenous TGFβ-1/SMAD3 signaling in macrophages (Liu et al., 2008). Others have reported that SOCS3 regulates IL-10 control of LPS-induced TNF, iNOS (inducible nitric oxide synthase) and nitric oxide (NO) in macrophages by targeting specific SOCS3 protein domains (SH2, SOCS box, and KIR) (Qasimi et al., 2006), thus associating SOCS3 with the IL-10 anti-inflammatory effects. SOCS3 inhibited STAT1 and regulated IFN-γ signaling, in response to LPS stimulation by binding to phosphorylated tyrosine sites of the JAK2 receptor domain (Stoiber et al., 1999) to control macrophage anti-bactericidal effects. Likewise, SOCS3 prevented IL-1 signaling, among others, by inactivating the TRAF-6/TAK1 complex (Posselt et al., 2011; Qin et al., 2012) to regulate LPS deleterious inflammatory responses.

Unlike SOCS1 and SOCS3, the control of LPS signaling by SOCS2 is minimal. Moreover, SOCS2 is differentially regulated in human and mouse cells (Frobose et al., 2006; Hu et al., 2009; Posselt et al., 2011), and the reason for this divergence has yet to be delineated. To promote TLR4 signaling, SOCS2 may target and mediate proteasome-dependent degradation of SOCS1 and SOCS3 (Tannahill et al., 2005; Hu et al., 2009). It is noteworthy to mention that some intact gram-negative organisms like *E. coli* (Qin et al., 2007; Hu et al., 2012; Demirel et al., 2013)*, P. aeruginosa* (Ding et al., 2017)*, Chlamydia pneumoniae* (Yang et al., 2008)*, Burkholderia pseudomallei* (Ekchariyawat et al., 2005)*, Salmonella enterica* (Uchiya and Nikai, 2005, 2008)*, Rickettsia conorii* (Colonne et al., 2013), and *Anaplasma phagocytophilum* (Bussmeyer et al., 2010) can directly stimulate the expression of SOCS1 and SOCS3 *in vitro* and *in vivo*. These organisms exploit multiple signaling pathways including STAT1, STAT3, MAPK and NF-κB to induce the transcription and/or protein expressions of SOCS1 or SOCS3 as a feedback mechanism to control their induced inflammatory responses (Ekchariyawat et al., 2005; Uchiya and Nikai, 2005, 2008; Yang et al., 2008; Bussmeyer et al., 2010; Colonne et al., 2013; Demirel et al., 2013; Ding et al., 2017).

### Gram-positive bacteria and SOCS proteins

**Figure 2:**
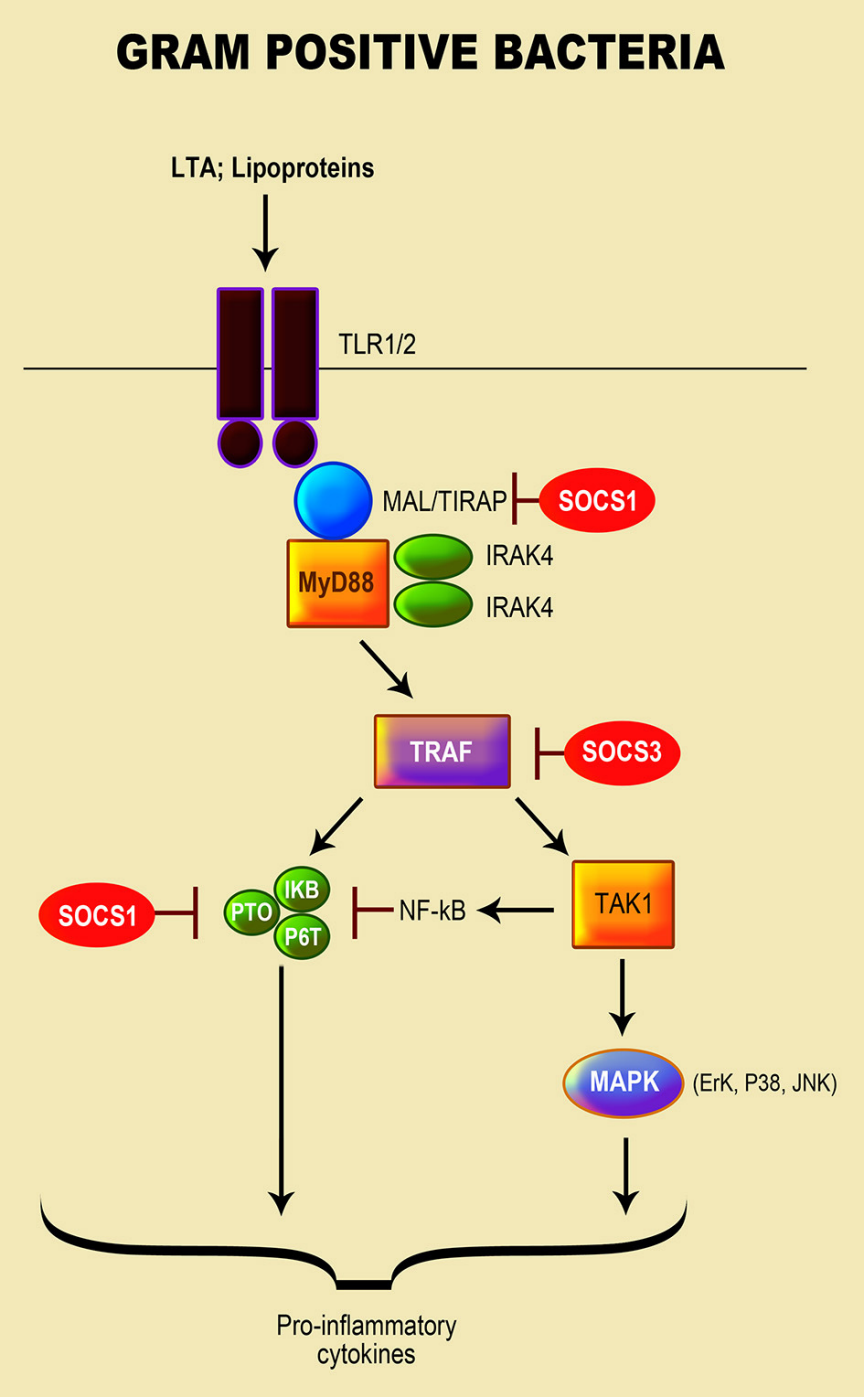
Role of SOCS proteins in the regulation of the signaling pathways induced by recognition of gram positive bacteria. Recognition of gram positive bacteria through their lipopeptide by TLR1 or/and 2. MyD88 and Toll/interleukin-1 (IL-1)-receptor (TIR)-domain mediates the activation of TLR2 by gram-positive bacteria, leading to the activation of the NF-κB pathway; MAPK signaling pathway via JNK, ERK-1, and p38 kinase activation; and pro-inflammatory caspase-1 (Schroder et al., 2003; Draing et al., 2008). The SOCS protein responsible for regulation of TLR signaling is highlighted in red.

Gram-positive bacteria such as *Listeria, Bacillus, Clostridium, Staphylococcus, Streptococcus*, and *Enterococcus* cause numerous severe infections in humans (Navarre and Schneewind, 1999; Plouffe, 2000; Hessle et al., 2005; Moellering, 2009; Woodford and Livermore, 2009; van 't Veer et al., 2011). These bacteria have a high resistance to a variety of antimicrobial therapies (Plouffe, 2000; Hessle et al., 2005; Moellering, 2009; Woodford and Livermore, 2009; van 't Veer et al., 2011; Schneewind and Missiakas, 2012) as their cell wall is composed of a layer of peptidoglycan (PGN) and lipoteichoic acid (LTA), encased in the cytoplasmic membrane by diacylglycerol (Nandi et al., 2004; Hessle et al., 2005; Brown et al., 2015). PGN is their principal PAMP that is recognized through Nod-like receptors [NLRs (Nod1 and Nod2)] and cryopyrin response proteins (Plouffe, 2000; Draing et al., 2008; Brown et al., 2015). Exposure to gram-positive bacteria triggers various patterns of pro-inflammatory cytokines notably, amongst many, IL-1α/β, TNF-α, IL-6, and IL-8 (Plouffe, 2000; Draing et al., 2008; Brown et al., 2015). TLR2 is the primary receptor activated in response to PGN and LTA (Draing et al., 2008). Furthermore, both *S. aureus* and *S. pneumoniae* LTA-recognition is attained by TLR2 associated with LBP and CD14 in human monocytes to y contribute in the pathogeneses of their diseases (McDonald et al., 2005). The activation of TLR2 by these bacteria is mediated by MyD88 and Toll/interleukin-1 (IL-1)-receptor (TIR)-domain, which leads to the activation of NF-κB, MAPK (via JNK, ERK-1, and p38) and pro-inflammatory caspase-1 (Schroder et al., 2003; Draing et al., 2008) (Figure 2).

The role of SOCS proteins in regulation of gram-positive bacteria-induced inflammation has not been extensively investigated in comparison to gram-negative bacteria and their LPS. Wu and colleagues (Son et al., 2015) reported that SOCS1 enhancement in macrophages infected with the pathogenic Group A *Streptococcus* (GAS), led to the blockage of cytokine expression. In addition to IFN-β signaling, which is involved in the GAS-induced SOCS1, the TLR4/MyD88 pathway was observed to play a crucial role in stimulating SOCS1 by forming a complex with JAK1/STAT1 (Son et al., 2015). Both *Bifidobacterium* (*B. breve, B. longum*, and *B. adolescentis*) and *E. faecalis* stimulated an increase in SOCS1 and SOCS3 mRNA transcripts in mouse macrophages by triggering NF-κB and MAPK signaling pathways to regulate the production of pro-inflammatory cytokines (Wu et al., 2015).

A study by Stoiber et al. (Okada et al., 2009) revealed that prolonged infection of macrophages with *L. monocytogenes* inhibited the phosphorylation of STAT1 and IFN-γ signaling with an enhancement of SOCS3 transcript and protein via the p38 MAPK pathway. Both live and heat-killed bacteria induced SOCS3; however, live bacteria induction of SOCS3 required de novo protein synthesis (Okada et al., 2009). The non-pathogenic probiotic bacterium *Lactobacillus* and non-pathogenic/pathogenic *Streptococcus* spp. induced the expression of SOCS3 mRNA in human primary macrophages by directly stimulating macrophages. Expression of SOCS3 by these bacteria was dependent on endogenously produced IL-10 and mediated through the p38 MAPK signaling pathway (Stoiber et al., 2001). Consequently, their stimulation of SOCS3 is induced directly, through at least p38 MAPK-mediated signaling pathway, and indirectly through IL-10 produced by bacterial-stimulated macrophages.

### Mycobacteria and SOCS proteins

The *Mycobacterium* genus includes, but not limited to, *M. tuberculosis* and *M. avium* complexes (Imai et al., 2003; Gao et al., 2006; Latvala et al., 2011), that are responsible for several pulmonary diseases in humans, in particular, Tuberculosis (TB) caused by *M. tuberculosis* (MTB) (Prince et al., 1989; Gao et al., 2006). Mycobacteria cell wall is composed of a thin internal layer of peptidoglycan, phosphatidyl-*myo*-inositol mannosides (PIMs) and arabinogalactan, and an external layer of hydrophobic mycolic acids (Nandi et al., 2004; Rottenberg and Carow, 2014). Other components include mannose-capped lipoarabinomannan (Man-LAM), a significant virulence factor; the related lipomannan (LM), and mannoglycoproteins (Rottenberg and Carow, 2014). Mycobacteria are facultative intracellular pathogens, and macrophages are their primary host cells (Gao et al., 2006; Kleinnijenhuis et al., 2011).

**Figure 3:**
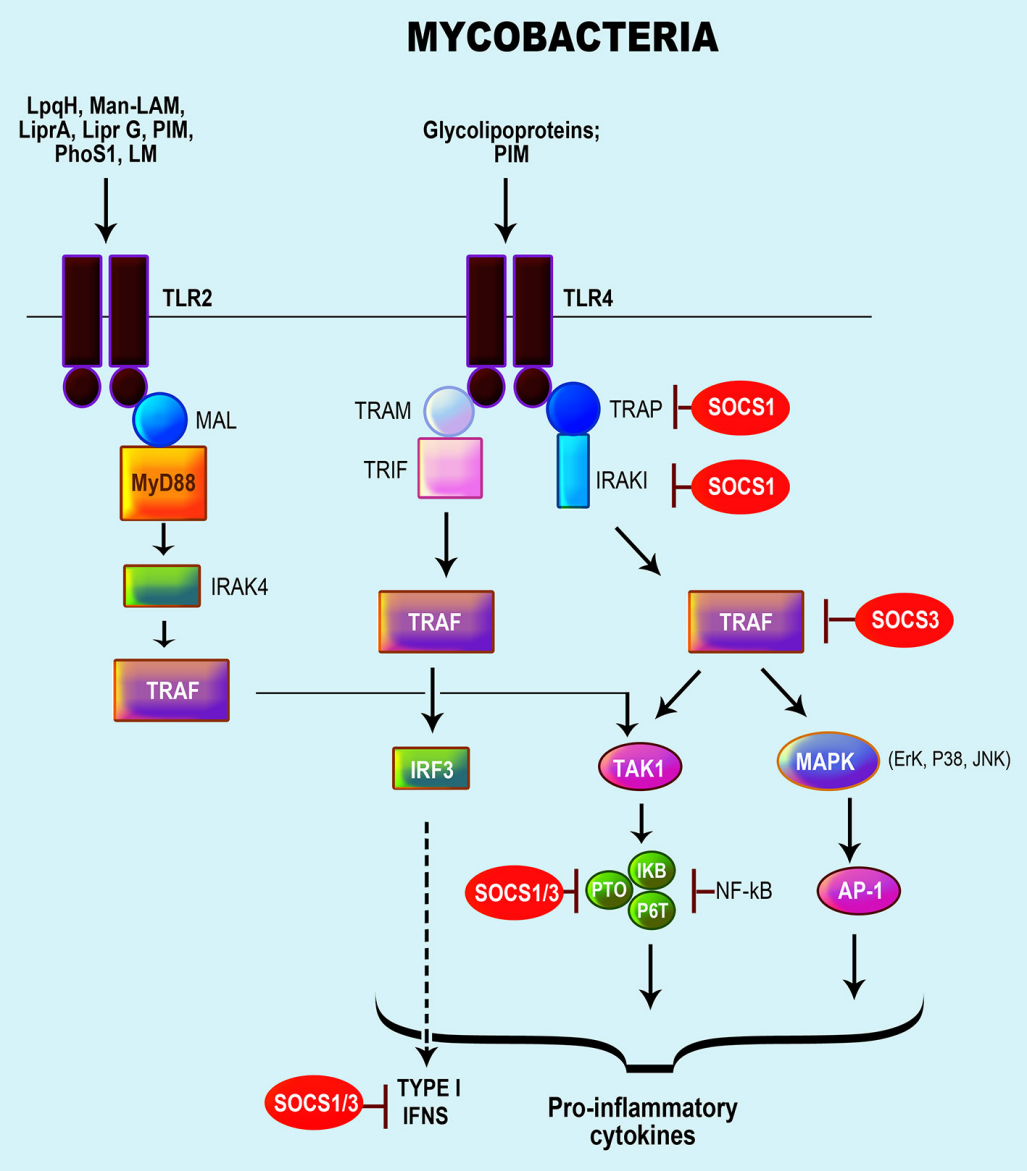
Role of SOCS proteins in the regulation of the signaling pathways induced by recognition of mycobacteria. Recognition of mycobacteria by the TLR 4 and TLR2. Mycobacteria activates the TLR 2/4 with a signaling cascade that results in the activation of NF-κB activated protein-1 (AP-1) via MyD88, MAL, and interleukin (IL)-1R-associated kinase (IRAK), resulting in the production of chemokines, pro-inflammatory cytokines particularly TNFα, IL-1β, IL-18, IL-12, and nitric oxide (Rajaram et al., 2014; Rottenberg and Carow, 2014; Zhao et al., 2014). Subsequently, the SOCS protein involved in the regulation of TLR signaling is accentuated in red.

Several PRRs are implicated in recognition of mycobacteria by host macrophages and DCs, including TLR1, TLR2, TLR4, and TLR9, C-type lectin receptors (CLRs) (i.e., mannose receptor, DC-SIGN, Mincle, and Dectin-1) and NLRs (Rottenberg and Carow, 2014; Zhao et al., 2014; Mortaz et al., 2015). Numerous mycobacterial components activate TLRs, namely lipoproteins (LpqH, LprA, LprG), PhoS1, LAM, LM, and PIMs, which activate TLR2; glycolipoprotein and PIM6, which activate TLR2/TLR4; and mycobacterial DNA, which respond via TLR9/TLR2 (Killick et al., 2013). Mycobacteria interaction with TLRs results in the activation of NF-κB activated protein-1 (AP-1) via MyD88, MAL, and IRAK, leading to the production of chemokines and several pro-inflammatory cytokines (Rajaram et al., 2014; Rottenberg and Carow, 2014; Zhao et al., 2014) (Figure 3). Mycobacterial components also induce IL-10 via caspase recruitment domain-containing protein 9 (CARD9) or p38 MAPK and serine/threonine Akt kinases (Jo, 2008; Redford et al., 2011).

Mycobacterial infections trigger the expressions of SOCS1 and SOCS3 (Gao et al., 2006; Dorhoi et al., 2010; Killick et al., 2013) along with SOCS4 and SOCS5 in mice infected with highly virulent MTB isolates (Vazquez et al., 2006). Overexpression of SOCS1 and SOCS3 results in polarizing effects permitting induction of suppressor responses, but also the survival of mycobacteria through the manipulation of cytokine responses, especially IFN-γ that is required in the resolution of mycobacterial infections (Manca et al., 2005; Dorhoi et al., 2010). Mycobacterial-induced SOCS1 and SOCS3 are dependent TLR2/MyD88 along with NF-κ and p38 MAPK activation (Manca et al., 2005). Specifically, SOCS1 suppressed STAT1 phosphorylation resulting in the inhibition of STAT1-mediated IFN-α/β signaling (Manca et al., 2005; Dorhoi et al., 2010). SOCS1 also promotes mycobacterial growth in macrophages by blocking IFN-γ secretion in response to IL-12 induced by the infection (Srivastava et al., 2009).

Mycobacteria-specific components (i.e., PIM2 and PPE protein, PPE-18) via TLR2/MyD88-activation of macrophages augment SOCS3 expression, and dislocation of the MyD88/TLR2 pathway modulated SOCS3 expression (Prince et al., 1989). Additionally, SOCS3 induced by PPE18 inhibited NF-κB activation by diminishing the phosphorylation of IκBα (Carow et al., 2011). In general, mycobacterial-induced SOCS3 inhibits STAT3 activation through cytokine receptors that activate STAT3 (Prince et al., 1989). As an example, SOCS3 binding to gp130 mediated the control of MTB infection in myeloid cells by inhibiting the IL-6/STAT3 signaling pathway (Nair et al., 2011; Carow et al., 2013).

Both SOCS2 and CIS can also play a role in regulating responses to mycobacterial infections. The expression of SOCS2 increased in macrophages infected with mycobacteria, and SOCS2-deficient mice exhibited a higher sensitivity to the inflammation induced by *M. bovis* infection (Carow and Rottenberg, 2014). Nonetheless, the activity of SOCS2 seems to be scarce and redundant and still requires a better understanding. CIS, on the other hand, is associated with increased susceptibility to TB (Sun et al., 2014; McCormick and Heller, 2015), likely by negatively regulating SOCS1 and SOCS3 (Trengove and Ward, 2013).

## Correlation of SOCS proteins with bacterial disease

Bacterial pathogens exploit SOCS proteins to manipulate cytokine receptor signaling and thereby influence infection outcomes as a strategy of evading host immune defenses (Baetz et al., 2007). Hence, the over-expression of SOCS proteins in bacterial infections supposedly is linked to the immune escape and exacerbation of disease. As SOCS1 and SOCS3 play essential roles in response to bacterial infections, they are therefore explicitly targeted for immune evasion. The reports above have therefore indicated that pathogens can induce SOCS1 and SOCS3 to evade deleterious host immune responses for their perpetuation and/or to control their induced inflammation. The most intended target is the interferon responses, mediated by STAT1 and controlled by SOCS1 and SOCS3, which play pivotal roles in the defense against bacterial infections.

The highly pathogenic bacterium, *L. monocytogenes* manipulates the macrophage machinery during early infection where there is heightened macrophage activation to permit its intracellular establishment. However, during persistent infections, *L. monocytogenes* regulates macrophage activation by inhibiting the transcription of IFN-γ and tyrosine phosphorylation of STAT1 via induction of SOCS3 (Okada et al., 2009). As stimulation of IFN-γ is necessary for macrophage activation and functions, inhibiting IFN-γ signaling is a stratagem utilized by *L. monocytogenes* to facilitate its intracellular survival by controlling its inflammation. Similarly, perturbations of IFN-γ and STAT1 signaling pathways by the facultative intracellular *B. pseudomallei* through induction of SOCS3 and CIS is a mechanistic tactic to reduce the macrophage bactericidal effect and enabled its intracellular survival (Ekchariyawat et al., 2005).

Results from studies by Uchiya and Nikai (Uchiya and Nikai, 2005, 2008) demonstrated how *Salmonella* pathogenicity island 2 (SPI-2) T3SS and its encoded virulence factor SpiC trigger SOCS3 up-regulation via the ERK1/2 pathway for inhibition of the JAK/STAT inflammatory signaling cascades for its continued survival in macrophages. GAS, which causes various systemic diseases induced SOCS1 that participates in the GAS' evasion of host immune responses in murine macrophages by dampening cytokine expression leading to rapid bacterial infection (Son et al., 2015). Expression of SOCS1 was shown to prevent *C. pneumoniae*-induced lethal inflammation through a STAT1 and IFN-α/β signaling-dependent manner, but conversely, its impact on IFN-α/β and IFN-γ impeded an efficient bacterial clearance (Yang et al., 2008). *Borrelia burgdoferi* (non-gram staining bacteria), the spirochetal agent of Lyme disease, stimulates the expression of SOCS1 and SOCS3 in macrophages to possibly control its inflammatory disorders (Khor et al., 2010). Additionally, *B. burgdorferi* via CD14 signaling induced SOCS1, SOCS3, and CIS as mediated by the p38 MAPK pathway to control the development of chronic inflammatory etiologies (Dennis et al., 2006).

Various mycobacteria manipulate IFN-γ-driven immunity by inducing SOCS1 and SOCS3 to evade the immune response or hamper the disease control. Augmentation of SOCS1 and SOCS3 levels and their subsequent inhibition of IFN-γ-induced STAT1 were found to alleviate the immune response for several mycobacterial species like *M. tuberculosis, M. avium*, and *M. bovis* (Gao et al., 2006; Srivastava et al., 2009; Dorhoi et al., 2010; Trengove and Ward, 2013). *M. bovis* infection stimulated SOCS1 and SOCS3 in mouse macrophages, which mediated the inhibition of IFN-γ-stimulated phosphorylation of STAT1 and thereby the subsequent inhibition of growth and activation of macrophages required for the control of this intracellular pathogen (Gao et al., 2006). Moreover, there are observations of both SOCS1 and SOCS3 association with disease progression in peripheral blood mononuclear cells and human macrophages of patients with TB (Sahay et al., 2009; Masood et al., 2012, 2013). SOCS1 and SOCS3 were up-regulated and contributed to Th2 immune polarization and down-modulation of Th1-mediated IFN-γ responses, and hence increased the disease severity by promoting the intracellular persistence of *M. tuberculosis* (Sahay et al., 2009; Masood et al., 2012, 2013). Infection of mice with highly virulent clinical isolates of MTB induced type I IFNs, which led to the up-regulation of SOCS1, SOCS4, SOCS5 and other negative regulators of the JAK/STAT pathway resulting in a decrease of Th1 type cytokines and decreased survival of MTB-infected mice (Vazquez et al., 2006).

## Implications and possible requirements for therapeutic approaches

SOCS proteins regulate cytokine signal transduction for maintaining immune functions but still contribute to the onset of immunological diseases and inflammation (Yoshimura et al., 2005). Therefore, modulating cytokine release holds promise for minimizing disease progression. SOCS1 and SOCS3 are tightly linked to cancer cell proliferation, as well as cancer-associated inflammation. In some cancer therapy studies, SOCS proteins have been used to control or suppress cytokine signaling for an efficacious treatment. One approach is overexpressing SOCS proteins to inhibit the growth of tumors mediated by suppressing tumor-promoting STATs. Another method is enhancing anti-tumor immunity by siRNA silencing of SOCS in DCs or CTLs (Ashenafi et al., 2014). In most cases, the silencing of SOCS1 and SOCS3 exacerbated carcinogenesis; thus, overexpression of SOCS1 and SOCS3 or SOCS-mimetics can be targeted therapeutics (Zhang et al., 2012). However, SOCS1 in DCs and likely T cells suppress anti-tumor immunity; therefore, silencing SOCS1 in these cells could also be therapeutic. Silencing of the SOCS1 gene may hinder the negative feedback regulation of the JAK/STAT pathway, therefore, resulting in heightened responsiveness to cytokines, and supporting survival and expansion of myeloma myeloid cells (Inagaki-Ohara et al., 2013). Blocking of constitutive STAT3 signaling results in growth inhibition and apoptosis of STAT3-positive tumor cells *in vitro* and *in vivo* (Galm et al., 2003). Development of SOCS-targeted therapeutics based on structural analysis of the JAK/SOCS complex (Zhang et al., 2012) could thus be a highly desirable approach.

The regulation of the levels of pro- and anti-inflammatory cytokines and chemokines by the immune system is critical in limiting or modulating the host defense against invading pathogens. SOCS proteins as negative regulators of JAK/STAT represent a promising target for anti-inflammatory therapies (Turkson and Jove, 2000). Therefore, the use of recombinant forms of SOCS proteins to refill the intracellular stores of SOCS needed to control acute or protracted inflammatory disease can be viewed as a novel targeted therapy to suppress the JAK/STAT pathway and prevent cytokine-mediated lethal inflammation (Recio et al., 2014). A recombinant cell-penetrating form of SOCS1 (CP-SOCS1) and SOCS3 were indeed shown to potently inhibit the JAK/STAT signaling pathway *in vitro* by interacting with the IFN-γ signaling complex and functionally reducing the phosphorylation of STAT1, which further resulted in inhibiting the production of pro-inflammatory cytokines and chemokines (Jo et al., 2005; DiGiandomenico et al., 2009; Fletcher et al., 2010). Moreover, CP-SOCS3 protected mice from lethal effects of Staphylococcal Enterotoxin B and LPS by decreasing the production of inflammatory cytokines (DiGiandomenico et al., 2009).

The exploitation of host SOCS proteins and manipulation of their functions by bacterial pathogens make them particularly attractive therapeutic targets. Therapeutic approaches targeting host-directed immunomodulatory components against bacterial infections have already been described (Finlay and Hancock, 2004; Hancock et al., 2012; Hawiger and Jo, 2013). Regulator peptides of the innate immune defense, agonists of innate immune receptors and adjuvants of innate immune components, have been tested for this purpose (Finlay and Hancock, 2004; Hawiger and Jo, 2013). TLRs and NOD receptors have, in fact, been targets of several immunomodulatory therapies, of which several are approved, to inhibit or treat bacterial infections (Finlay and Hancock, 2004; Hancock and Sahl, 2006). Among them, the most notable example is CADI-05, an agonist for many TLRs, which was successfully investigated as a potential therapy for TB (Finlay and Hancock, 2004; Hancock and Sahl, 2006). Besides, vaccines formulated with small molecule immune-potentiators that trigger TLRs were shown efficient in protection against bacterial infections. A notable example is the vaccine adjuvant based on a TLR7 agonist adsorbed to alum (Alum-TLR7), which induced a high and broad protection against *Staphylococcus aureus* (Hennessy et al., 2010; Bagnoli et al., 2015). Furthermore, targeting STAT activity that is strictly regulated by SOCS1 and SOCS3 proteins, by inhibiting tyrosine kinases, could allow avoiding the subversion of the innate immune responses during a bacterial infection and therefore represents an attractive antibacterial therapeutic approach. For instance, dual-inhibitors of Ser/Thr protein kinases PknG/PknG, which are required for mycobacteria growth were able to prevent their replication in mice (Hennessy et al., 2010; Gil et al., 2013; Mancini et al., 2016). However, investigations of SOCS proteins as therapeutic targets have not been beyond the development of a cell-penetrating form of SOCS to compensate the loss of endogenous SOCS (DiGiandomenico et al., 2009; Recio et al., 2014). Nonetheless, since bacteria target several intracellular pathways, many of which are linked to the SOCS proteins, it is clear that SOCS proteins represent unnavigable therapeutic targets in the control or eradication of bacterial infections.

## Conclusion and future directions

A better understanding of the control mechanisms involved in SOCS modulation of immune responses and inflammation is key to developing effective targeted therapeutics and vaccines. Each SOCS protein contributes either to the negative regulation of cytokine signaling or the regulation of many biological processes. The expression of SOCS proteins can define host susceptibility to infection by facilitating accelerated bacterial growth or protecting the host from severe inflammation. However, the direct action of the SOCS-mediated inhibition in inflammatory response is yet to be fully elucidated, thus limiting the progress being made scientifically and clinically by microbiologist and immunologists. Additionally, factors such as increased virulence, mutations and antibiotic resistance over time pose recurring challenges in the control of epidemic bacterial diseases worldwide. Admittedly, SOCS-targeted therapy for bacterial-induced inflammation is provocative, but yet one that should be considered exploring. So far, inhibiting the action of JAKs by small composites or drugs show reparative potential (DiGiandomenico et al., 2009). Such insight along with advances in medicine and technology may offer more efficient, and novel strategies surrounding SOCS therapy to control the inflammatory bacterial disease.

## Author contributions

SD did the literature search and exploration and wrote the manuscript. DB and RS helped with writing and reading the manuscript. SS read and edited the manuscript. VD edited the manuscript and coordinated the project. All authors read and approved the final manuscript.

### Conflict of interest statement

The authors declare that the research was conducted in the absence of any commercial or financial relationships that could be construed as a potential conflict of interest.
